# Cellular and molecular pathways linking obesity to skeletal muscle dysfunction

**DOI:** 10.1007/s11033-026-12146-6

**Published:** 2026-06-23

**Authors:** Diego Pinheiro, Anabelle Silva Cornachione

**Affiliations:** Laboratory of Muscle Physiology and Biophysics, Department of Physiological Sciences, Federal University of São Carlos (UFSCar), São Carlos, SP Brazil

**Keywords:** Obesity, Skeletal muscle, Metabolic inflammation, Lipotoxicity, Insulin resistance, Myokines

## Abstract

Obesity is increasingly recognized as a condition that directly impairs skeletal muscle structure, metabolism, and endocrine function through complex molecular and cellular mechanisms extending beyond the classical concept of sarcopenic obesity. This narrative review aimed to synthesize current evidence regarding the intracellular signaling pathways, metabolic alterations, and endocrine interactions involved in obesity-induced skeletal muscle dysfunction independent of overt sarcopenia. Relevant literature from experimental, clinical, and review studies was identified through searches of PubMed, Scopus, and Web of Science databases, focusing on obesity-associated alterations in skeletal muscle metabolism, ectopic lipid accumulation, inflammatory signaling, mitochondrial dysfunction, and adipose–muscle crosstalk. Current evidence indicates that obesity per se promotes skeletal muscle dysfunction through ectopic lipid deposition, lipotoxicity, mitochondrial impairment, and chronic low-grade inflammation mediated by dysregulated intracellular signaling pathways. Altered adipomyokine signaling, including interleukin-6 and tumor necrosis factor-α, further contributes to impaired insulin signaling, reduced metabolic flexibility, oxidative stress, and compromised muscle integrity. These molecular and cellular alterations reinforce skeletal muscle as both a target and an active regulator of obesity-associated metabolic inflammation. Collectively, these findings support the concept that obesity intrinsically disrupts skeletal muscle metabolic and endocrine homeostasis independently of sarcopenic obesity and highlight the importance of targeted strategies aimed at preserving skeletal muscle metabolic function and overall metabolic health.

## Introduction

Obesity is a multifactorial metabolic disease of epidemic proportions, characterized by a body mass index (BMI) above 30 kg/m². Its global prevalence has continued to rise in recent decades, leading to a substantial impact on public health and to an increased burden of chronic diseases such as type 2 diabetes, metabolic dysfunction–associated fatty liver disease, cardiovascular diseases, some types of cancer, and impairments in skeletal muscle. All of these outcomes are driven by excess body fat resulting from a long-term imbalance between energy intake and expenditure [1–4].

Skeletal muscle accounts for 40% to 50% of body weight and absorbs up to 75% of circulating glucose, in addition to its important role in fatty acid oxidation; therefore, it is a primary target in the therapy of diabetes, metabolic syndrome, and other diseases. In obesity, fat increases within and between muscle fibers, which may favor insulin resistance. When intramyocellular fat accumulation becomes chronic, adverse metabolic effects are observed, such as mitochondrial dysfunction and reduced protein synthesis mediated by lipotoxicity [5–6]. Excessive expansion of adipose tissue can cause systemic and muscular complications through chronic low-grade inflammation, as well as through migration of immune system cells and secretion of pro-inflammatory adipokines [7–8].

When analyzing the scientific literature on obesity and skeletal muscle, it becomes apparent that most studies address sarcopenic obesity, traditionally characterized by the coexistence of excess adiposity and loss of muscle mass [9–11]. Although this phenomenon is relevant, it captures only part of the interaction between obesity and skeletal muscle. There is a conceptual gap in the literature regarding the role of obesity per se (independent of classical sarcopenia) on metabolic and structural pathways in skeletal muscle across different population contexts, including young adults and individuals with diabetes and lipid disorders. This gap compromises an integrated understanding of the cellular and systemic mechanisms involved, such as the crosstalk between muscle and adipose tissue mediated by adipokines and myokines, as well as the impact of prolonged inflammatory responses on muscle function [12–13].

The present narrative review aims to critically synthesize the recent literature on the mechanisms by which obesity influences skeletal muscle, exploring metabolic, inflammatory, and endocrine components that go beyond the simple definition of sarcopenic obesity, and proposing an integrative model that considers both molecular impacts and clinical implications. This is a critical narrative review, carefully structured to integrate conceptual evidence and recent pathophysiological mechanisms that elucidate the relationship between obesity and changes in skeletal muscle. Unlike systematic reviews or meta-analyses, this work did not strictly follow PRISMA criteria, since the primary objective was to develop an integrative theoretical and analytical framework rather than to estimate a quantitative effect size.

Study selection was performed in the PubMed/MEDLINE, Scopus, Web of Science, and Google Scholar databases, with complementary searches in the reference lists of key articles. Search terms included combinations of keywords such as: obesity, skeletal muscle, lipotoxicity, inflammation, myokines, metabolic dysfunction, sarcopenic obesity, insulin resistance, and their Portuguese equivalents. To maximize thematic and contemporary relevance, articles published over the last 25 years were prioritized, with special emphasis on publications addressing molecular mechanisms and clinical implications.

Inclusion criteria comprised critical and narrative reviews on obesity and muscle tissue; experimental and clinical studies investigating structural and functional changes in skeletal muscle associated with obesity; articles addressing metabolic interactions between adipose tissue and muscle (adipo-myokines and myokines); and studies discussing chronic low-grade inflammation, insulin resistance, and mitochondrial adaptations. Exclusion criteria comprised articles dealing exclusively with pharmacological therapies without a direct relationship to skeletal muscle; studies narrowly focused on specific disease conditions without systemic relevance (for example, isolated cancer without metabolic discussion); and opinion publications without empirical support.

The literature was then critically analyzed to identify recurring conceptual patterns, knowledge gaps, and potential future research directions, with special attention to the most recent contributions that expand the understanding of obesity–muscle mechanisms beyond the strict definition of sarcopenic obesity.

## Skeletal muscle and adipose tissue in obesity

Adipose tissue may accumulate in three main body regions: visceral, subcutaneous, and ectopic depots. Ectopic fat accumulation, which is highly prevalent in obesity, corresponds to the deposition of adipose tissue within internal organs, skeletal muscle, and other non-adipose compartments. These sites are not physiologically specialized for lipid storage and, therefore, ectopic lipid deposition is strongly associated with metabolic dysfunction [14–15].

Fat deposited in skeletal muscle can be classified into three types: intermuscular fat, located between muscle groups; intramuscular fat, composed of adipocytes situated between muscle fibers; and intramyocellular lipids, corresponding to lipid droplets present within myocytes [16, 17]. Adipocytes located between fibers and between muscle groups represent fat depots situated beneath the muscle fascia and are structurally and metabolically distinct from subcutaneous adipose tissue [18].

### Intramyocellular lipid deposition and lipotoxicity

Intramyocellular lipid (IMCL) depots correspond to the accumulation of lipids within myofibrils and skeletal muscle fibers. These lipids are predominantly stored as triacylglycerol (TAG) within lipid droplets; however, several bioactive lipid intermediates, including diacylglycerols (DAG), ceramides, acylcarnitines, and sphingolipids, also accumulate in skeletal muscle during obesity and are strongly associated with metabolic dysfunction [16, 19, 45–47]. Importantly, neutral lipid storage per se does not necessarily impair insulin sensitivity, as endurance-trained athletes may also exhibit elevated IMCL content while maintaining preserved metabolic flexibility and insulin responsiveness, a phenomenon commonly referred to as the “athlete’s paradox” [45]. Therefore, the metabolic consequences of IMCL accumulation appear to depend not only on the total lipid content, but also on the intracellular localization, lipid species composition, oxidative capacity, and mitochondrial function of skeletal muscle fibers.

Among the different lipid intermediates, DAG and ceramides are considered key mediators of obesity-induced skeletal muscle insulin resistance. Increased intramyocellular DAG levels promote the activation of novel protein kinase C (PKC) isoforms, particularly PKCθ, leading to serine phosphorylation of insulin receptor substrate-1 (IRS-1) and impairment of downstream PI3K/Akt insulin signaling pathways. Consequently, insulin-stimulated GLUT4 translocation and glucose uptake are reduced, contributing to insulin resistance [47]. Similarly, ceramide accumulation directly inhibits Akt phosphorylation and disrupts insulin signaling, while also promoting mitochondrial dysfunction, oxidative stress, and pro-inflammatory signaling pathways [47].

The magnitude of intramyocellular lipid accumulation also appears to depend on muscle fiber type. Evidence from experimental obesity models indicates that fast-twitch glycolytic fibers, such as those present in the gastrocnemius and intermediate plantar muscles, exhibit greater lipid droplet accumulation compared with predominantly oxidative slow-twitch muscles such as the soleus [6, 16]. This difference may be partially explained by the higher oxidative capacity and mitochondrial density of type I fibers, which favor lipid oxidation and reduce the accumulation of lipotoxic intermediates.

In obesity, excessive lipid oversupply frequently exceeds the oxidative capacity of skeletal muscle, resulting in incomplete fatty acid oxidation, accumulation of reactive lipid species, and increased mitochondrial stress. Recent evidence demonstrates that obesity impairs mitochondrial adaptations related to substrate oxidation, electron transport chain efficiency, reactive oxygen species (ROS) handling, and metabolic flexibility [13]. These alterations compromise cellular energetic homeostasis and reinforce mitochondrial dysfunction as a central mechanism linking obesity to skeletal muscle metabolic deterioration.

## Mitochondrial dysfunction in obesity-associated skeletal muscle impairment

Mitochondrial dysfunction has emerged as a central mechanism linking obesity to skeletal muscle metabolic impairment. In physiological conditions, skeletal muscle mitochondria play a crucial role in substrate oxidation, ATP production, metabolic flexibility, and reactive oxygen species (ROS) homeostasis. However, chronic lipid oversupply and nutrient excess in obesity frequently exceed the oxidative capacity of skeletal muscle, resulting in mitochondrial stress, impaired bioenergetics, and progressive metabolic deterioration [13, 48].

One of the earliest mitochondrial alterations observed in obesity is the reduction in oxidative capacity and fatty acid oxidation efficiency. Excessive lipid availability promotes incomplete β-oxidation and accumulation of intermediate lipid metabolites, including acylcarnitines and ceramides, which contribute to mitochondrial overload and insulin resistance [47, 48]. In parallel, obesity is associated with reduced expression of genes involved in mitochondrial biogenesis and oxidative metabolism, particularly peroxisome proliferator-activated receptor gamma coactivator-1 alpha (PGC-1α), a key regulator of mitochondrial function and oxidative phenotype in skeletal muscle [49]. Reduced PGC-1α activity impairs mitochondrial biogenesis, decreases oxidative phosphorylation capacity, and compromises metabolic function.

Obesity-associated mitochondrial dysfunction also involves alterations in electron transport chain (ETC) activity and coupling efficiency. Defects in ETC complexes reduce ATP synthesis efficiency and increase electron leakage, contributing to excessive ROS production [48, 50]. Although physiological ROS generation participates in cellular signaling and adaptive responses, chronic ROS overproduction promotes oxidative damage to mitochondrial proteins, lipids, and DNA, exacerbating mitochondrial dysfunction and impairing skeletal muscle insulin signaling pathways. Increased oxidative stress may also activate stress-sensitive inflammatory pathways, including c-Jun N-terminal kinase (JNK) and nuclear factor kappa B (NF-κB), reinforcing chronic low-grade inflammation within skeletal muscle [54].

In addition to bioenergetic defects, obesity alters mitochondrial quality control mechanisms, including mitochondrial dynamics and mitophagy. Balanced mitochondrial fusion and fission are essential for maintaining mitochondrial integrity and cellular energetic homeostasis. Experimental evidence suggests that obesity disrupts the expression of proteins involved in mitochondrial fusion, such as mitofusin-2 (Mfn2), while favoring excessive mitochondrial fragmentation and impaired mitochondrial turnover [51]. Defective mitophagy contributes to the accumulation of dysfunctional mitochondria, further increasing ROS generation and metabolic stress.

Another important consequence of mitochondrial dysfunction in obesity is the loss of metabolic flexibility, defined as the ability of skeletal muscle to appropriately switch between lipid and glucose oxidation according to nutrient availability and energetic demands. Obese individuals frequently exhibit impaired substrate switching and reduced oxidative adaptability, contributing to insulin resistance and diminished metabolic efficiency [52]. This metabolic inflexibility appears to be strongly associated with mitochondrial dysfunction, lipid overload, and chronic inflammatory signaling [58].

Collectively, these findings demonstrate that mitochondrial dysfunction in obesity extends beyond a simple reduction in oxidative capacity. Instead, it involves a complex network of alterations affecting substrate oxidation, ROS homeostasis, mitochondrial quality control, inflammatory signaling, and metabolic flexibility. These mechanisms reinforce the central role of mitochondria in obesity-associated skeletal muscle dysfunction and highlight mitochondrial pathways as potential therapeutic targets for preserving skeletal muscle metabolic health.

## Metabolic properties of intra- and intermuscular fat

The accumulation of intra- and intermuscular fat is associated with reduced muscle mass, decreased strength, and impaired insulin sensitivity. Intra-/intermuscular adipose tissue is functionally similar to visceral adipose tissue, particularly regarding its ability to induce inflammation and negatively influence insulin signaling pathways [8, 18]. Notably, comparative analyses of the composition of inflammatory proteins secreted by intra/intermuscular, visceral, and subcutaneous fat deposits in obese individuals demonstrate that intra/intermuscular fat has a greater capacity to release pro-inflammatory cytokines and chemokines than visceral and subcutaneous fat [4, 18].

Furthermore, intra-/intermuscular fat can elevate interstitial free fatty acids concentrations, leading to metabolically compromised muscular microenvironment and myocytes insulin resistance [4, 8]. This reduced sensitivity may contribute directly to the decline in muscle mass and strength observed in obesity [5, 10].

## Other cellular types and inter-/intramuscular fat

In addition to fibro-adipogenic progenitor cells, other cellular types play an important role in the accumulation of inter- and intramuscular fat, including skeletal muscle satellite cells and adipose tissue–derived stem cells, which may migrate from adipose tissue to skeletal muscle. Satellite cells are essential for muscle regeneration and growth. However, in vitro studies have demonstrated that these cells may differentiate into adipocytes in the presence of adipogenic stimuli [13].

Furthermore, intramuscular preadipocytes impair satellite cell differentiation through activation of the c-Jun N-terminal kinase (JNK)/mitogen-activated protein kinase (MAPK) signaling pathways. Concomitantly, fat deposition is facilitated through activation of the peroxisome proliferator-activated receptor (PPAR), reinforcing adipogenic commitment within the skeletal muscle microenvironment [13].

A third cellular population capable of contributing to inter- and intramuscular fat deposition consists of bone marrow–derived mesenchymal stem cells. Although their contribution has been described, the precise mechanisms and factors that promote their migration and differentiation within skeletal muscle tissue remain incompletely understood and require further investigation [17].

## Adipomyokines

**Fig. 1:**
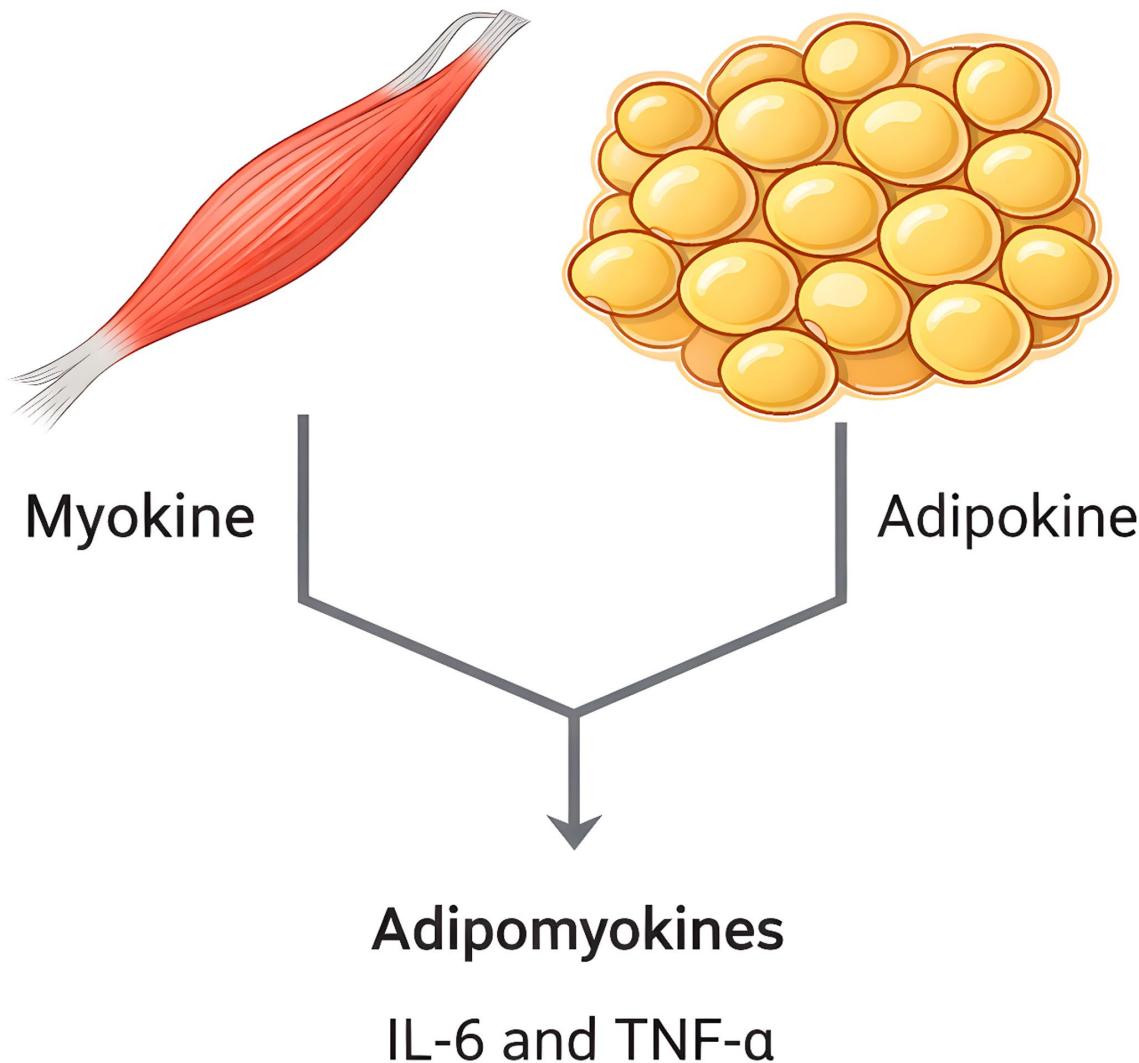
Schematic representation of the bidirectional endocrine crosstalk between skeletal muscle and adipose tissue. Skeletal muscle secretes myokines, whereas adipose tissue releases adipokines. Some signaling molecules, such as interleukin-6 (IL-6) and tumor necrosis factor-α (TNF-α), can be produced by both tissues and are therefore classified as adipomyokines, highlighting the shared and integrated role of muscle and adipose tissue in metabolic and inflammatory regulation. Figures were prepared using Canva Pro and Microsoft PowerPoint

Several studies have demonstrated that a considerable number of myokines and adipokines are capable of exerting overlapping biological functions. These molecules may be secreted by both skeletal muscle and adipose tissue, reinforcing the concept of bidirectional endocrine communication between these organs [11, 20]. Among the most extensively studied adipomyokines are interleukin-6 (IL-6) and tumor necrosis factor-α (TNF-α), which will be discussed in detail below (Fig. 1) [4, 5].

### IL-6

Interleukin-6 (IL-6) is secreted by several tissues in the body, including skeletal muscle, adipose tissue, and immune cells. As a myokine, IL-6 plays a key role in muscular performance during contraction. Conversely, when chronically secreted as an adipokine, it may contribute to the development of insulin resistance [4, 11].

IL-6 exerts its biological effects by binding to its receptor (IL-6R), thereby activating Janus-activated kinase (JAK) and downstream transcriptional signaling pathways. The biological role of IL-6 in vivo remains controversial, as it may exert both anti-inflammatory and pro-inflammatory effects depending on the physiological and metabolic context [20].

Acting as a metabolic sensor, IL-6 plays an important role in energy metabolism. Glycogen depletion increases IL-6 gene and protein expression, indicating its involvement in the regulation of energy pathways. When released in an endocrine manner by both type I and type II muscle fibers, IL-6 acts through the gp130Rβ/IL-6Rα receptor complex, subsequently activating AMPK and/or PI3K pathways, which enhance glucose uptake and stimulate fatty acid oxidation [19, 4].

It is noteworthy that IL-6 expression occurs predominantly in type I muscle fibers, whereas type II fibers primarily express TNF-α [18]. IL-6 deficiency increases fatty acid transporter levels and intramuscular lipid content in type I fibers, but not in type II fibers, in experimental animal models [16].

In adipose tissue, elevated IL-6 concentrations increase lipolysis and fatty acid oxidation via AMPK activation and modulate leptin expression. Experimental silencing of IL-6 reduces inguinal adipose tissue mass and decreases GLUT-4 concentration, AMPK phosphorylation, and fatty acid synthase mRNA expression in this tissue. Muscle-derived IL-6 may therefore modulate adipose tissue metabolism by increasing glucose uptake and regulating lipolytic and lipogenic factors [4, 11].

### TNF-α

Tumor necrosis factor-α (TNF-α) is a cytokine secreted by adipose tissue and is strongly involved in the development of insulin resistance in adipocytes [3, 8]. However, TNF-α is also secreted by several other cell types, including skeletal muscle cells [11, 18]. In vitro experiments using muscle cells demonstrate that exposure to TNF-α inhibits myoblast differentiation and impairs insulin-like growth factor-1 (IGF-1) signaling [21].

Through activation of TNF receptor 1 (TNFR1), TNF-α reduces AMPK activity, thereby attenuating acetyl-CoA carboxylase phosphorylation and fatty acid oxidation. This favors increased intramuscular diacylglycerol accumulation, contributing to skeletal muscle insulin resistance [22]. Conversely, TNF-α may limit adipose tissue expansion by optimizing glucose metabolism, accelerating lactate production, and increasing lipolysis in adipocytes via direct paracrine action [3]. Additionally, the adipomyokine TNF-α plays an important role in angiogenic and potentially regenerative activity in adipose tissue–derived stem cells. TNF-α stimulation increases cell proliferation, F-actin microfilament formation, motility, migration through the extracellular matrix, and the expression of pro-angiogenic factor mRNA [23].

## Inflammatory signaling in obesity-associated skeletal muscle dysfunction

Although adipose tissue has traditionally been considered the primary site of chronic low-grade inflammation in obesity, growing evidence demonstrates that skeletal muscle also actively participates in obesity-associated inflammatory signaling [24, 25]. In obesity, skeletal muscle is not merely a passive target of systemic inflammation, but rather an active immunometabolic organ capable of producing cytokines, recruiting immune cells, and amplifying local inflammatory responses. These alterations contribute directly to insulin resistance, mitochondrial dysfunction, and metabolic inflexibility.

Skeletal muscle myocytes can express and secrete several cytokines, chemokines, and regulatory peptides, including interleukin-6 (IL-6), IL-8, IL-15, fibroblast growth factor 21 (FGF21), irisin, myonectin, and myostatin [26–29]. The secretion profile of these molecules is highly context dependent. Under physiological conditions, particularly during acute exercise, myokines exert anti-inflammatory and metabolically beneficial effects, promoting glucose uptake, lipid oxidation, and metabolic adaptation [27, 29]. In contrast, chronic nutrient excess and obesity favor persistent activation of inflammatory signaling pathways, resulting in increased production of pro-inflammatory mediators within skeletal muscle.

One of the central mechanisms linking obesity to skeletal muscle inflammation involves excessive free fatty acid (FFA) exposure and lipid overflow. Saturated fatty acids may activate Toll-like receptor 4 (TLR4) signaling in myocytes and resident immune cells, leading to activation of nuclear factor kappa B (NF-κB) and c-Jun N-terminal kinase (JNK) pathways [53, 56]. These signaling cascades increase the transcription of pro-inflammatory cytokines, including TNF-α, IL-6, and monocyte chemoattractant protein-1 (MCP-1), thereby amplifying inflammatory responses within skeletal muscle [54]. Importantly, activation of JNK and NF-κB pathways also impairs insulin signaling through serine phosphorylation of insulin receptor substrate-1 (IRS-1), contributing to skeletal muscle insulin resistance.

In addition to intrinsic myocyte inflammatory signaling, obesity promotes infiltration of immune cells into skeletal muscle, particularly macrophages and T lymphocytes [24, 33]. Experimental studies demonstrate that high-fat diets increase macrophage accumulation within skeletal muscle even before overt systemic metabolic dysfunction becomes evident [33]. Histologically, these immune cells are frequently localized within intermuscular adipose tissue or adjacent to muscle fibers, creating a local inflammatory niche characterized by elevated cytokine production and oxidative stress [34, 35].

Intermuscular adipose tissue itself appears to play an important immunometabolic role in obesity-associated skeletal muscle dysfunction. Unlike subcutaneous adipose tissue, intermuscular fat depots exhibit a highly inflammatory phenotype and are strongly associated with insulin resistance and systemic inflammatory markers [34]. Expansion of intermuscular adipose tissue promotes local secretion of cytokines, chemokines, and adipokines, facilitating immune cell recruitment and maintenance of chronic low-grade inflammation within skeletal muscle microenvironments [18, 34]. This inflammatory milieu may further exacerbate mitochondrial dysfunction, impair substrate oxidation, and reduce metabolic flexibility.

Among inflammatory mediators, TNF-α represents one of the most extensively studied cytokines in obesity-associated skeletal muscle dysfunction. Cultured myocytes derived from obese individuals with insulin resistance or type 2 diabetes exhibit increased secretion of TNF-α and MCP-1 compared with non-obese individuals [30]. Elevated TNF-α levels may impair mitochondrial function, suppress AMPK activity, reduce fatty acid oxidation, and exacerbate insulin resistance [22, 24]. Similarly, IL-6 signaling appears to exert dual and context-dependent effects. Acute IL-6 release during exercise promotes AMPK activation and metabolic adaptation, whereas chronically elevated IL-6 levels in obesity are associated with persistent inflammatory signaling and impaired insulin sensitivity [29, 55, 6, 56].

**Fig. 2:**
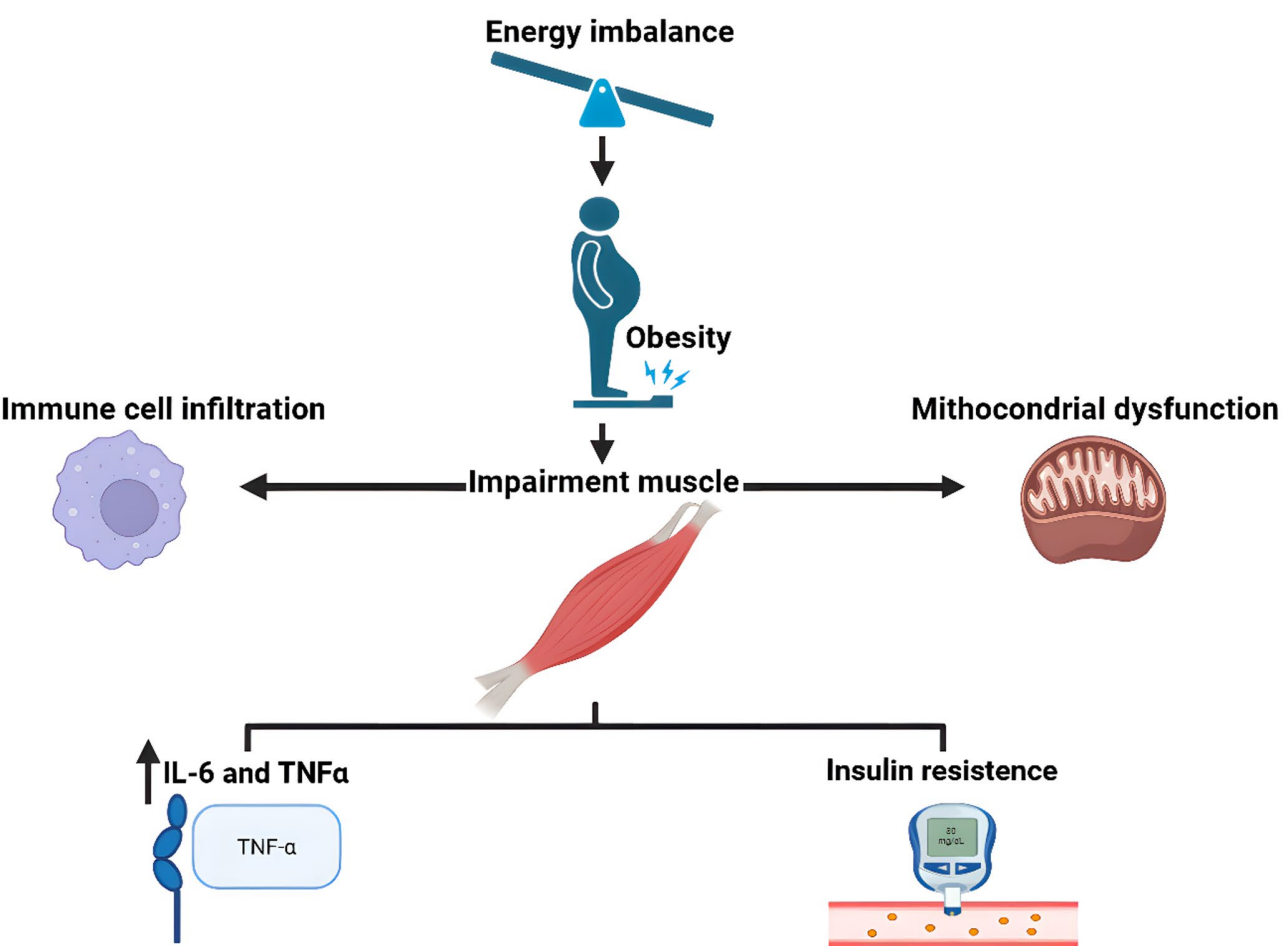
Conceptual model illustrating the impact of energy imbalance–induced obesity on skeletal muscle function. Obesity is associated with skeletal muscle impairment, which is characterized by immune cell infiltration, increased expression of inflammatory mediators such as interleukin-6 (IL-6) and tumor necrosis factor-α (TNF-α), mitochondrial dysfunction, and the development of insulin resistance. Together, these alterations contribute to metabolic and inflammatory dysfunction within skeletal muscle in obesity. Figures were prepared using Canva Pro and Microsoft PowerPoint

Collectively, these findings indicate that skeletal muscle inflammation in obesity results from complex interactions among myocytes, infiltrating immune cells, intermuscular adipose tissue, and inflammatory signaling pathways. This integrated inflammatory network contributes directly to insulin resistance, mitochondrial dysfunction, and metabolic deterioration in obesity-associated skeletal muscle impairment (Fig. 2).

## Myostatin in obesity

Myostatin was the first molecule secreted by skeletal muscle to be identified and classified as a myokine. This protein is detectable in the circulation following its secretion by muscle tissue. Myostatin belongs to the transforming growth factor-β (TGF-β) superfamily, and inactivation of the gene encoding myostatin results in marked skeletal muscle hypertrophy in rodents, livestock, and humans [36].

Myostatin plays a central role in regulating skeletal muscle growth and is also involved in maintaining metabolic homeostasis and modulating adipose tissue function and mass. Experimental observations in myostatin-deficient mice have demonstrated increased muscle mass accompanied by reduced adipose tissue accumulation [36, 37]. In both humans and animal models, aerobic and resistance training attenuate myostatin expression, and genetic or pharmacological inhibition of myostatin appears to potentiate the metabolic benefits of exercise, including improvements in insulin sensitivity and body composition [37, 38].

**Fig. 3:**
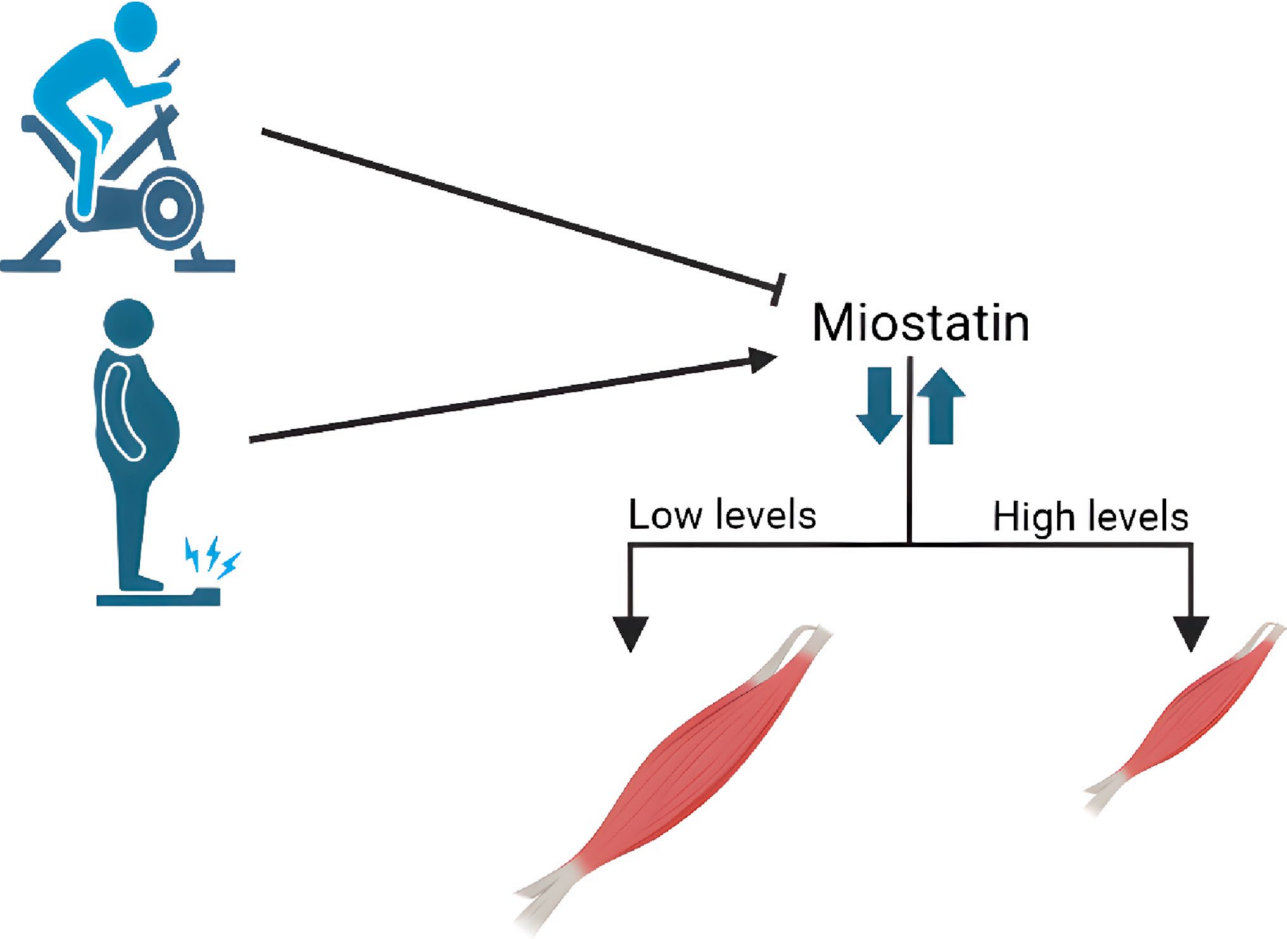
Schematic representation of myostatin regulation and its impact on skeletal muscle mass. Different physiological and metabolic conditions, such as physical exercise and obesity, modulate myostatin levels. Low myostatin levels are associated with increased skeletal muscle mass, whereas high myostatin levels are linked to reduced muscle mass, highlighting the central role of myostatin in the regulation of muscle size. Figures were prepared using Canva Pro and Microsoft PowerPoint

A growing body of evidence supports the association between elevated myostatin expression and obesity. Increased circulating and intramuscular myostatin levels have been observed in individuals with obesity (Fig. 3) [39]. Furthermore, myostatin derived from myotubes isolated from skeletal muscle biopsies exhibits higher expression levels in obese women compared with non-obese controls [39]. These findings suggest that excessive myostatin activity may contribute to reduced muscle mass and metabolic impairment in obesity.

Follistatin, another member of the TGF-β superfamily, is classified as a hepatokine rather than a myokine and acts as a natural inhibitor of myostatin, playing a critical role in skeletal muscle hypertrophy. Elevated plasma follistatin levels are observed in healthy individuals following acute exercise sessions. In animal models subjected to acute swimming exercise, increased circulating follistatin levels and elevated hepatic follistatin mRNA expression have been reported [40]. This systemic rise in follistatin may contribute to the regulation of skeletal muscle myostatin expression in response to exercise.

Thus, follistatin represents a key mediator of the liver–muscle crosstalk during and after physical activity [40]. Through follistatin-mediated inhibition of myostatin, exercise may reduce the elevated myostatin levels observed in obesity and mitigate its deleterious effects on skeletal muscle mass and metabolic function [31].

## Obesity-associated skeletal muscle dysfunction and sarcopenic obesity: shared and distinct mechanisms

Sarcopenic obesity represents a complex condition characterized by the coexistence of excess adiposity and impaired skeletal muscle quantity and function [32, 41]. Although traditionally associated with aging, sarcopenic obesity shares several molecular and metabolic mechanisms with obesity-associated skeletal muscle dysfunction, including insulin resistance, chronic low-grade inflammation, mitochondrial dysfunction, oxidative stress, and ectopic lipid accumulation. However, additional age-related mechanisms, such as anabolic resistance, hormonal decline, and impaired regenerative capacity, may further exacerbate skeletal muscle deterioration in older individuals [10, 11, 41, 42].

The pathogenesis of sarcopenic obesity is multifactorial and involves interactions among physical inactivity, poor dietary habits, chronic inflammation, oxidative stress, and metabolic dysfunction [11]. Similar to obesity-associated skeletal muscle impairment, intramuscular lipid accumulation and chronic exposure to inflammatory mediators contribute to insulin resistance and mitochondrial dysfunction within myocytes. Excessive lipid accumulation may impair mitochondrial β-oxidation, promote reactive oxygen species (ROS) generation, and favor the accumulation of lipotoxic intermediates, thereby reinforcing inflammatory signaling and metabolic inflexibility [11]. Consequently, skeletal muscle becomes progressively more susceptible to functional decline, proteolysis, and reduced regenerative capacity.

Importantly, several mechanisms overlap between obesity-associated skeletal muscle dysfunction and sarcopenic obesity. Both conditions exhibit chronic activation of inflammatory pathways, increased expression of cytokines such as TNF-α and interleukins, impaired insulin signaling, oxidative stress, and altered adipomyokine secretion [10, 11, 43, 44]. Moreover, mitochondrial dysfunction and metabolic inflexibility appear to represent central pathophysiological mechanisms in both conditions, contributing to reduced substrate oxidation efficiency and impaired skeletal muscle metabolic homeostasis.

Nevertheless, sarcopenic obesity also presents distinct characteristics strongly associated with aging. Age-related anabolic resistance reduces skeletal muscle protein synthesis responsiveness to dietary amino acids and exercise stimuli, thereby impairing muscle maintenance [41]. In addition, aging is associated with progressive reductions in physical activity levels, satellite cell regenerative potential, neuromuscular integrity, and endocrine function. In men, age-related hypogonadism may negatively affect skeletal muscle and adipose tissue regulation, whereas menopause-related estrogen decline may contribute to altered adipose tissue distribution and skeletal muscle dysfunction in women [41]. Additional endocrine disorders, including growth hormone deficiency and Cushing’s syndrome, may further aggravate these alterations.

Nutritional factors also appear to contribute significantly to the progression of sarcopenic obesity. Inadequate protein intake may compromise skeletal muscle maintenance and accelerate functional decline, while low vitamin D levels may negatively affect muscle performance and metabolic health [36, 38, 39]. Furthermore, altered expression of myokines involved in muscle mass regulation, particularly increased myostatin and reduced irisin levels, may contribute to impaired muscle remodeling and reduced lean mass preservation [40, 42].

**Fig. 4:**
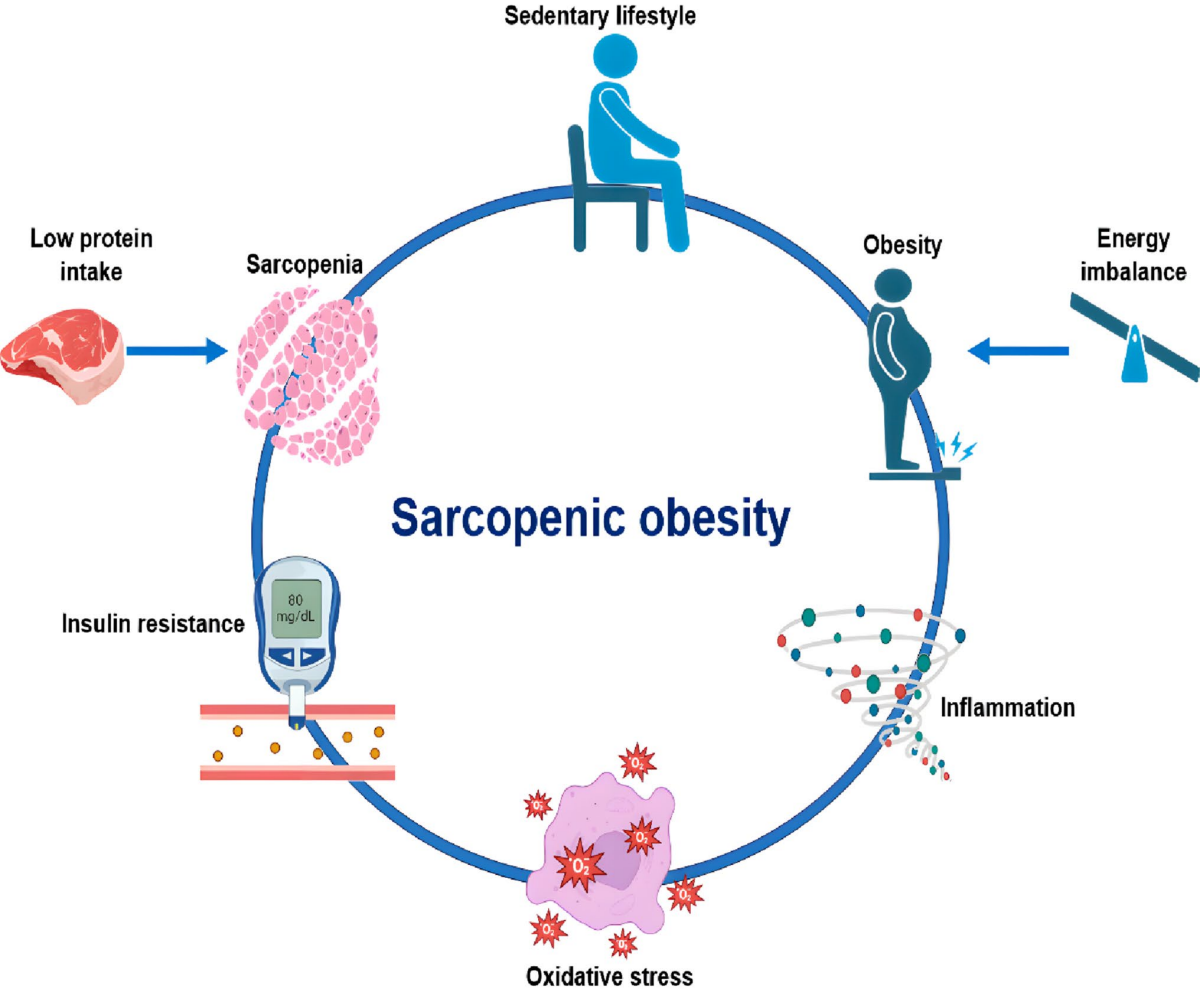
Conceptual model illustrating the multifactorial and self-perpetuating nature of sarcopenic obesity. Sedentary lifestyle and energy imbalance contribute to the development of obesity, which is associated with chronic inflammation, oxidative stress, and insulin resistance. Concurrently, low protein intake and metabolic disturbances promote sarcopenia. These interconnected mechanisms reinforce each other, establishing a vicious cycle that sustains and aggravates sarcopenic obesity. Figures were prepared using Canva Pro and Microsoft PowerPoint

From a clinical and translational perspective, preservation of skeletal muscle mass and metabolic function should be considered a central component in obesity management strategies. Emerging evidence suggests that interventions aimed at maintaining skeletal muscle integrity may improve insulin sensitivity, metabolic flexibility, and systemic inflammatory regulation [34, 35, 57]. Therefore, although sarcopenic obesity shares several mechanistic pathways with obesity-associated skeletal muscle dysfunction, aging-related anabolic and endocrine alterations likely intensify skeletal muscle deterioration and contribute to the progression of this complex metabolic condition (Fig. 4).

## Final considerations

Obesity should no longer be viewed solely as a disorder of excessive adipose tissue accumulation, but rather as a complex systemic condition capable of profoundly altering skeletal muscle metabolic and endocrine homeostasis. The current evidence demonstrates that obesity per se promotes skeletal muscle dysfunction through integrated mechanisms involving ectopic lipid deposition, lipotoxicity, mitochondrial dysfunction, chronic low-grade inflammation, impaired insulin signaling, and dysregulated adipomyokine secretion. These alterations compromise not only skeletal muscle metabolic flexibility and energetic efficiency, but also the endocrine and immunometabolic functions of muscle tissue itself.

Importantly, obesity-associated skeletal muscle dysfunction appears to occur independently of classical sarcopenic obesity. Although several pathophysiological mechanisms overlap between these conditions, obesity alone is capable of inducing substantial metabolic and molecular disturbances within skeletal muscle even in the absence of overt muscle mass loss. This distinction has important clinical implications, as metabolically impaired skeletal muscle may already be present in younger individuals and in earlier stages of obesity before the development of clinically detectable sarcopenia.

The mechanisms underlying obesity-induced skeletal muscle dysfunction involve complex bidirectional interactions among myocytes, immune cells, adipose tissue, and intracellular signaling pathways. Chronic lipid oversupply promotes the accumulation of lipotoxic intermediates such as diacylglycerols and ceramides, contributing to mitochondrial overload, oxidative stress, and activation of inflammatory pathways including NF-κB and JNK signaling. Simultaneously, alterations in adipomyokine secretion and persistent inflammatory activation further impair insulin signaling and substrate oxidation, establishing a self-perpetuating cycle of metabolic deterioration within skeletal muscle.

Nevertheless, several important questions remain unresolved. The temporal progression of obesity-induced skeletal muscle dysfunction remains incompletely understood, particularly regarding the differential effects of short-term versus long-term obesity exposure. In addition, relatively little is known about the reversibility of these alterations following substantial weight loss or metabolic recovery in previously obese individuals. Another important limitation of the current literature is the predominance of experimental animal models, which may not fully reproduce the complexity and heterogeneity of obesity-associated skeletal muscle dysfunction in humans. Factors such as aging, sex differences, physical activity levels, dietary patterns, and obesity duration likely modulate skeletal muscle responses and should be more consistently explored in future studies.

From a translational perspective, preservation of skeletal muscle metabolic integrity should be considered a central therapeutic target in obesity management. Interventions capable of improving mitochondrial function, metabolic flexibility, and skeletal muscle insulin sensitivity particularly physical exercise, nutritional optimization, and strategies aimed at reducing chronic inflammation may play a critical role in attenuating obesity-associated metabolic dysfunction. Future investigations integrating molecular, metabolic, and clinical approaches will be essential to clarify the mechanisms underlying obesity- induced skeletal muscle impairment and to support the development of targeted therapeutic strategies focused on preserving skeletal muscle health in obesity.

## Data Availability

No datasets were generated or analysed during the current study.
